# Caregiver burden in amyotrophic lateral sclerosis is more dependent on patients’ behavioral changes than physical disability: a comparative study

**DOI:** 10.1186/1471-2377-12-156

**Published:** 2012-12-07

**Authors:** Patricia Lillo, Eneida Mioshi, John R Hodges

**Affiliations:** 1 Neuroscience Research Australia, Sydney, Australia; 2 School of Medical Sciences, University of New South Wales, Sydney, Australia; 3 Departamento de Neurología Sur. Facultad de Medicina, Universidad de Chile, Santiago, Chile

**Keywords:** ALS, Behavioral changes, FTD, Caregiver burden

## Abstract

**Background:**

Behavioral changes in patients with amyotrophic lateral sclerosis (ALS) mirror those found in frontotemporal dementia (FTD). Considering the high rate of neuropsychiatric symptoms found in ALS patients, this paper examines whether caregiver burden is associated with behavioral changes *over and above* the physical disability of patients with ALS, and if the presence of caregivers’ depression, anxiety and stress also impacts on caregiver burden.

**Methods:**

140 caregivers of patients with ALS participated in a postal survey investigating patients’ neuropsychiatric symptoms (Cambridge Behaviour Inventory Revised CBI-R), motor function (Amyotrophic Lateral Sclerosis Functional Rating Scale Revised - ALSFRS-R), caregiver burden (Zarit Burden Interview), and caregiver mood (Depression, Anxiety and Stress Scale- DASS21). Seventy four percent of them were caregivers of patients with limb onset and 25.7% were caregivers of patients with bulbar onset.

**Results:**

Moderate to severe behavioral changes were reported in 10-40% of patients with ALS. The levels of depression, anxiety and stress in the caregivers reached 20%. Burden was high in 48% of the caregivers. The strongest predictor of high caregiver burden was ALS patients’ abnormal behavior rather than physical disability, with an odds ratio of 1.4, followed by caregivers’ stress.

**Conclusions:**

Our study has identified that behavioral changes (e.g. disinhibition, impulsivity) and caregiver stress have greater impact on caregiver burden than level and pattern of physical disability.

## Background

Over the past decade there has been increasing awareness of behavioral changes in patients with amyotrophic lateral sclerosis (ALS), which mirror those found in frontotemporal dementia (FTD) [1,2]. Caregivers of patients with FTD present with higher levels of burden than those caring for Alzheimer’s disease (AD) patients [3,4]. Caregiver burden seems to be closely coupled with the high rate of behavioral changes in FTD, [5-7] although this finding is not universal [8,9].

Caregiver burden is a complex construct involving the caregiver’s feelings about the emotional/physical health, social life and financial status that they suffer as a result of caring for their relatives [10]. Caregiving was shown to have an adverse effect on the physical and psychological health of caregivers [11]. Although advancing ALS undoubtedly causes high levels of caregiver distress, few studies have focused on the contributory factors [12-14]. Caregiver burden, depression and anxiety appear inter-related, and associated with the ALS patients’ physical disability [15]. Also, neurobehavioral symptoms have a negative impact on caregivers’ psychological status [16]. Given the high rate of cognitive deficits and neuropsychiatric symptoms found in ALS patients [2,17,18], it is clearly germane to examine whether caregiver burden is associated with behavioral changes *over and above* the physical disability of patients with ALS.

The aims of this study were: 1) to assess the impact of ALS patients’ behavior and motor disability on caregiver burden, 2) to assess the frequency of depression, anxiety and stress of the caregivers and possible impact on caregiver burden.

## Methods

Caregivers of patients with ALS were contacted through the Motor Neurone Disease Association Australia, who kept their identities anonymous. Of 354 ALS patients registered as members of the association in NSW, 114 of their caregivers (32%) accepted an invitation to participate in a postal survey evaluating neuropsychiatric symptoms and motor function in ALS patients and burden of the caregiver. Additionally, 56 caregivers from other Australian states accepted the invitation through the local MND associations. Of the final one hundred and seventy caregivers who agreed to participate, 30 (17.6%) of them did not continue the study due to patients’ health issues or personal problems. Finally, data from the caregivers of 140 ALS patients was collected (60% from NSW and 40% from other Australian states); 104 (74.3%) of them were caregivers of patients with limb onset, whereas 36 (25.7%) were caregivers of patients with bulbar onset. Ethics approval was obtained from the Human Research Ethics Committee of South Eastern Sydney/Illawarra Area Health Service. Signed consent was obtained from each participant.

### Patient - motor function

A self- administered version of the Amyotrophic Lateral Sclerosis Functional Rating Scale Revised (ALSFRS-R) was completed by ALS patients and/or their caregivers. It contains 4 sub scores: bulbar, fine, gross and respiratory function [19,20]. A total score of 48 denotes normal function.

### Patient - behavioral changes

Changes in ALS patients’ behaviour were measured with the Cambridge Behavioural Inventory Revised (CBI-R), a caregiver-based measure of neuropsychiatric symptoms and daily activities, which correlates highly with the Neuropsychiatric Inventory (NPI).

It contains 10 domains: memory, everyday skills, self care, mood, beliefs, sleep, eating habits (e.g. preference for sweet food, increased appetite), abnormal behavior (e.g. disinhibition and impulsivity), stereotypic and/or motor behaviours and lack of motivation (apathy) [21]. The behavioral changes assessed by the CBI-R are independent of motor function and depression [2]. Scores were converted into percentage of impairment: 0-25% was classified as mild, 26-50% as moderate, 51-75% as severe, and > 75% as very severe [2,22].

### Caregiver - depression, anxiety and stress scale

Caregivers completed the Depression, Anxiety, Stress scale (DASS 21) [23]. The following cut offs were applied: depression > 9, anxiety > 7 and stress > 14. The degree of severity for all sub-scales was classified as follows: normal, mild, moderate, severe and extremely severe.

### Caregiver burden

The Zarit Burden Interview (ZBI) was used to measure caregiver burden by evaluating disease impact on caregivers’ quality of life, psychological suffering and impact on social and family relationships. Caregivers completed a self-administered short version of 12 items, which has shown to have comparable psychometric properties to the full version. The ZBI has a 0–4 point score with a maximum score of 48. A score ≥17 indicates a high burden [10,24,25].

#### Data analyses

SPSS Statistics 17.0 was used for analysis of results. To examine clinical features reported by caregivers, independent *t*-tests or Mann Whitney *U* tests were used where appropriate.

Comparison of behavioral changes across motor onset groups was analysed using Pearson’s *X*^2^ tests with Yates’ correction for continuity and Fisher’s exact test. A logistic regression was used to analyse the impact of caregiver’s mood, patients’ motor function, and behavioral changes on caregiver burden.

## Results

The caregiver group (n = 140) was predominantly composed by females (69.3%). The mean age was 60.8 ± 12 years. The majority of caregivers were the patient’s spouses (90%), followed by patient’s children (7%); other relatives or friends were occasionally caregivers (3%).

ALS patients’ mean age was 63.5 ± 9 years, with a median of disease duration of 3 years (interquartile range 2–5). The mean ALSFRS-R total score was 30.4 (S.D. 9.7). Distribution of patients by motor onset showed a predominance of limb (73.6%) over bulbar (26.4%) onset. The age range and pattern of disease onset (limb vs. bulbar) mirrors closely that found in large cohorts [26-28].

### Motor function

Comparison of ALSFRS-R total scores between patients with limb and bulbar onset showed no significant difference (*p* > 0.1). As expected, patients with bulbar onset had lower scores on bulbar function than patients with limb onset (limb Md = 9, bulbar Md = 4, U = 411, Z = −6.7, *p* < 0.001, r = 0.6). Likewise, patients with limb onset had lower scores in fine motor (limb Md = 6, bulbar Md = 9, U = 995.5, Z = −3.8, *p* < 0.001, r = 0.3) and gross motor function (limb Md = 6, bulbar Md = 8, U = 1171, Z = −2.9, *p* = 0.004, r = 0.2). No significant difference was found for respiratory function across groups (limb Md = 10, bulbar Md = 9, *p* > 0.1).

### Behavioral changes

**Figure 1:**
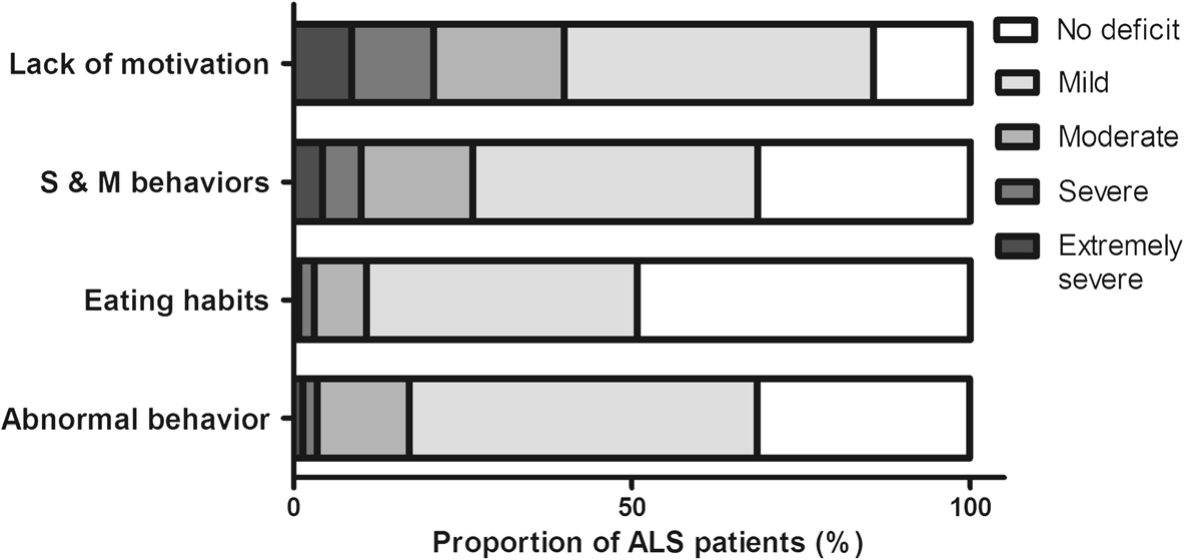
**Behavioral changes in ALS patients based on CBI-R caregiver reports.** (S & M behaviors: stereotypic and motor behaviors).

Figure 1 shows that abnormal behavior, stereotypical and motor behaviors and changes in eating habits were reported in more than 50% of the ALS patients, with around 10–30% patients in the moderate-severe category. The most prominent feature was lack of motivation (apathy), reported in more than 80% of the cases, with 40% having moderate-severe apathy.

Comparison of CBI-R scores across patients with limb and bulbar onset showed no significant association between motor onset and behavioral changes (*p* > 0.1).

### Depression, anxiety and stress

**Figure 2:**
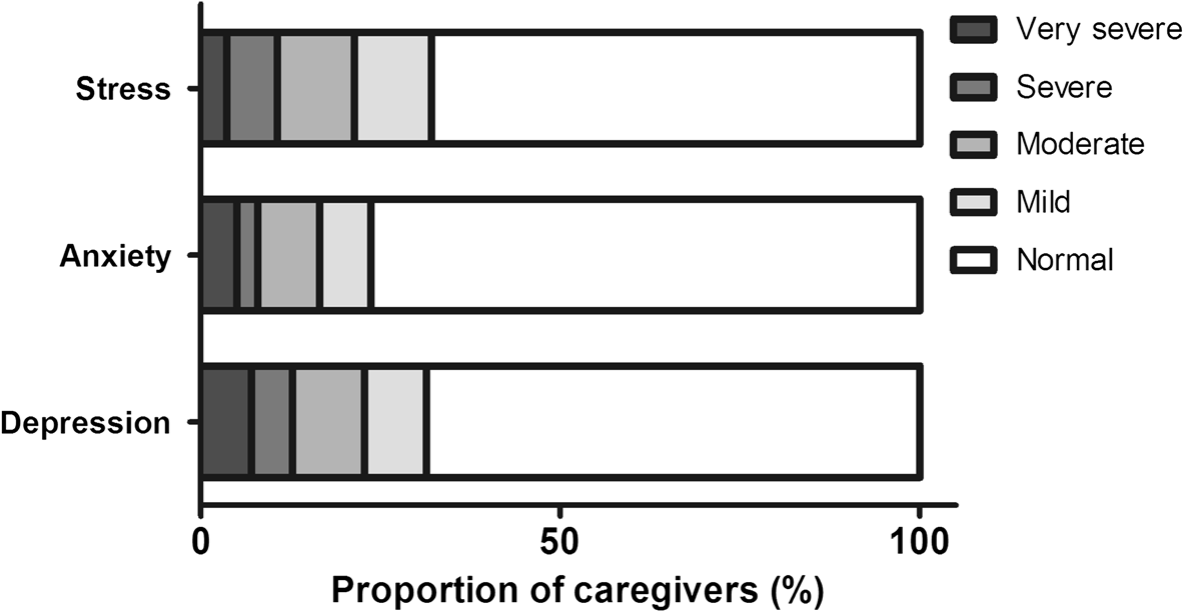
Depression, anxiety and stress based on caregiver self report (DASS21).

As shown in Figure 2, 69% of the caregivers did not reach the threshold for depression on the DASS. Moderate to very severe depression was present in 23% of the caregivers, and lesser degrees of depression were reported by 9% of caregivers.

The percentage of caregivers with anxiety and stress was lower. Overall, 16% had moderate to extremely severe anxiety and 21% equivalent levels of stress. Comparison of the results between caregivers of patients with limb and bulbar onset showed no significant association between motor onset and presence of depression, anxiety and stress (*p* > 0.1).

### Caregiver burden

The Zarit Burden Interview showed that high levels of burden (score ≥ 17) were reported by 48% of the caregivers. There was no significant difference when comparing caregivers’ burden of patients with limb and bulbar onset (*p* > 0.1).

Logistic regression was performed to assess the impact of different factors on the likelihood of caregivers in presenting with high burden. The patient related variables considered were motor function (*ALSFRS*-*R sub scores*: bulbar, fine motor, gross motor and respiratory function) and behavioral changes (*CBI*-*R domains*: abnormal behavior, eating habits, stereotypical and abnormal behavior and apathy). Caregivers’ variables considered were depression, anxiety and stress (*DASS 21* sub scales).

The full model containing all predictors was statistically significant, (*X*^2^ [*11*, *N* = *140*] = *74*.*1 p* < *0*.*001*), and explained between 45% (Cox and Snell R Square) and 60% (Nagelkerke R squared) of the variance in caregiver burden scores. Of the caregivers predicted to have high levels of burden, the model correctly classified 83.1%. As shown in Table 1, only two independent variables made statistically significant contributions to the model; abnormal behavior (from the patient) and stress (from the caregiver). The strongest predictor of high burden was abnormal behavior with an odds ratio of 1.4. This indicated that caregivers of ALS patients with abnormal behavior were 1.4 times more likely to report high burden.

**Table 1:** Logistic regression: variables predicting likelihood of high burden on caregivers of patients with ALS

**Variable**	**B**	**S.E.**	**Wald**	**df**	**p**	**O.R.**	**95% CI for O.R.**	
							**Lower**	**Upper**
Abnormal behavior (CBI-R)	0.37	0.13	8.36	1	0.004*	1.44	1.13	1.85
Eating habits (CBI-R)	0.02	0.17	0.02	1	0.894	1.02	0.74	1.41
Stereotypic & motor behaviors (CBI-R)	0.10	0.11	0.79	1	0.376	1.10	0.89	1.36
Motivation (CBI-R)	0.04	0.06	0.34	1	0.555	1.04	0.92	1.18
Depression (DASS)	0.06	0.06	1.36	1	0.243	1.07	0.96	1.19
Anxiety (DASS)	−0.08	0.04	3.88	1	0.051	0.93	0.86	1.00
Stress (DASS)	0.12	0.052	5.10	1	0.024*	1.12	1.02	1.25
Bulbar (ALSFRS-R)	−0.07	0.08	0.76	1	0.384	0.93	0.79	1.1
Fine motor (ALSFRS-R)	−0.14	0.10	1.97	1	0.167	0.87	0.71	1.06
Gross motor (ALSFRS-R)	0.79	0.11	0.50	1	0.478	1.08	0.87	1.35
Respiratory (ALSFRS-R)	−0.00	0.11	0.00	1	0.975	0.10	0.80	1.24
Constant	−2.11	1.22	2.99	1	0.84	0.121		

The caregiver burden was explained predominantly by the presence of abnormal behavior (in patients) and stress (in caregivers). * = statistically significant.

## Discussion

Our study has identified that behavioral changes (e.g. disinhibition, impulsivity) and caregivers’ level of stress have greater impact on caregiver burden than any other patient variables, including level and pattern of physical disability.

Behavioral symptoms, as measured by the Frontal Systems Behavioral Scale (FrSBe), have previously been reported to be associated to higher caregiver burden, [16,18] although only one study has considered variables related to caregivers [16]. We have confirmed this association using the Cambridge Behavioural Inventory - Revised and, importantly, found that the strongest predictor of caregiver burden was the patient’s abnormal behavior. It should be noted that, as in other studies, apathy was the most common behavioral change [2,18,29], but this factor did not enter the model of caregiver burden. Stereotypical behaviors were also reported at similar levels to abnormal behavior, but again, this factor did not explain caregiver burden. Specific changes in behavior, notably disinhibition and impulsivity, are particularly distressing for caregivers. We suspect that many clinicians involved in the management of ALS patients are unaware of the high rate of these changes and their impact on family relationships. Specific interventions targeting carers and developing their coping skills have been shown to be successful in FTD [30] and may have a successful place in the management of ALS dyads.

A number of studies have shown that caregivers of patients with ALS report high levels of burden, anxiety and depression in association with the patient’s loss of physical functions [14,15]. In our study, somewhat surprisingly, burden was not associated with changes in physical function, as measured by the ALSFRS-R. One possible explanation for this lack of association may be that behavioral changes which overshadow the lesser influence of physical disability in a log regression model. Moderate or worse depressive symptoms were present in 23% of caregivers, with similar rates of stress. These symptoms did not, however, enter the model explaining caregiver burden. The reported rate of caregiver depression in caregivers of people with ALS has varied from 11 to 61% [31,32] which almost certainly reflects the use of different instruments, the threshold used to determine depression and the level of disability of patients in the cohorts. It is interesting that caregiver depression had lesser role in the genesis of caregiver burden than seen in pure FTD, despite the overlap in behavioral symptoms between bvFTD and ALS [12,13].

The levels of anxiety and stress (around 20%) in our caregivers were lower than reported previously [33,34]. Again, the variability probably reflects the use of different instruments. Caregivers’ state anxiety has been previously related to their trait anxiety more than external factors [34]. Stress rather than depression and anxiety was the main psychological caregiver variable contributing to caregiver burden. This is clearly relevant to management since can be addressed in MND clinics and are potentially amenable to intervention.

A longitudinal study identified that, initially, caregiver distress was best predicted by the psychosocial impact of ALS symptoms and emotional lability. Later, caregiver distress related to satisfaction with social relationships and social support [13] rather than behavioral changes, although these were limited to dysexecutive symptoms rather than the FTD-like features explored by the CBI. Future longitudinal studies should investigate the role of specific behavioral changes, caregiver variables, and disease progression especially given the fact that caregiver burden seems to increase over time. A limitation of this study was the nature of the survey via the MND Association, where carers who responded to the invitation to the research study were likely to be those willing to respond the questionnaires. Still, our results corroborated those published previously, and the patients’ characteristics mirrored those in large cohorts.

The fact that ALS patients presented similar behavioural symptoms to patients with FTD has been reinforced by a recent genetic discovery. The presence of a hexanucleotide repeats in the intron of C90RF72 in almost a quarter of cases with familial ALS and 12% of the cases with familial FTD [35,36] support strongly the idea of the continuum between ALS and FTD.

## Conclusions

In summary, behavioral changes appear to lead to considerable caregiver burden, highlighting the importance to screen for neuropsychiatric symptoms in MND Clinics. Importantly, these symptoms could be modified by psychological or pharmaceutical therapies in an attempt to reduce caregiver burden. Caregivers’ emotional state also affects burden, with stress (but not depression or anxiety) playing major roles. Professionals involved with the care of ALS patients should be aware of these specific caregivers’ needs, which once addressed, are also likely to benefit the wellbeing of the patients.

## Competing interests

Dr Lillo is supported by a CONICYT scholarship (Government of Chile) and Faculty of Medicine, University of Chile.

Dr. Mioshi serves on the editorial board of *Dementia and Geriatric Cognitive Disorders *and is a recipient of a National Health and Medical Research Council of Australia Early Career Fellowship and an Motor Neurone Disease Research Institute of Australia Grant.

Prof Hodges is funded by the Australian Research Council Federation Fellowship, FF0776229.

Prof. Hodges serves on editorial boards of *Aphasiology*, *Cognitive Neuropsychiatry*, and *Cognitive Neuropsychology*; receives royalties from publication of *Cognitive Assessment for Clinicians* (Oxford University Press, 2007) and *Frontotemporal Dementia Syndromes* (Cambridge University Press, 2007); and receives fellowship support from the Australian Research Council Federation.

## Authors’ contributions

In order to give appropriate credit to each author of a paper, the individual contributions of authors to the manuscript should be specified in this section. PL: 1) have made substantial contributions to conception and design, acquisition of data, analysis and interpretation of data; 2) have been involved in drafting the manuscript and revising it critically for important intellectual content; and 3) have given final approval of the version to be published. EM: 1) have made substantial contributions to conception and design, analysis and interpretation of data; 2) have been involved in revising it critically for important intellectual content; and 3) have given final approval of the version to be published. JRH: 1) have made substantial contributions to conception and design, analysis and interpretation of data; 2) have been involved in revising it critically for important intellectual content; and 3) have given final approval of the version to be published.

## Pre-publication history

The pre-publication history for this paper can be accessed here:

http://www.biomedcentral.com/1471-2377/12/156/prepub

## References

[B1] Merrilees J Klapper J Murphy J Lomen-Hoerth C Miller BL Cognitive and behavioral challenges in caring for patients with frontotemporal dementia and amyotrophic lateral sclerosis Amyotroph Lateral Scler 2010 11 3 298 302 10.3109/17482961003605788 20222805 PMC2908374

[B2] Lillo P Mioshi E Zoing MC Kiernan MC Hodges JR How common are behavioural changes in amyotrophic lateral sclerosis? Amyotroph Lateral Scler 2011 12 1 45 51 10.3109/17482968.2010.520718 20849323

[B3] Riedijk SR De Vugt ME Duivenvoorden HJ Niermeijer MF Van Swieten JC Verhey FR Tibben A Caregiver burden, health-related quality of life and coping in dementia caregivers: a comparison of frontotemporal dementia and Alzheimer’s disease Dement Geriatr Cogn Disord 2006 22 5–6 405 412 16966830 10.1159/000095750

[B4] Kaiser S Panegyres PK The psychosocial impact of young onset dementia on spouses Am J Alzheimers Dis Other Demen 2006 21 6 398 402 17267371 10.1177/1533317506293259

[B5] Mourik JC Rosso SM Niermeijer MF Duivenvoorden HJ Van Swieten JC Tibben A Frontotemporal dementia: behavioral symptoms and caregiver distress Dement Geriatr Cogn Disord 2004 18 3–4 299 306 15305107 10.1159/000080123

[B6] Knutson KM Zamboni G Tierney MC Grafman J Neural correlates of caregiver burden in cortical basal syndrome and frontotemporal dementia Dement Geriatr Cogn Disord 2008 26 5 467 474 10.1159/000167268 18984957 PMC2596937

[B7] Boutoleau-Bretonniere C Vercelletto M Volteau C Renou P Lamy E Zarit burden inventory and activities of daily living in the behavioral variant of frontotemporal dementia Dement Geriatr Cogn Disord 2008 25 3 272 277 10.1159/000117394 18285675

[B8] Riedijk S Duivenvoorden H Rosso S Van Swieten J Niermeijer M Tibben A Frontotemporal dementia: change of familial caregiver burden and partner relation in a Dutch cohort of 63 patients Dement Geriatr Cogn Disord 2008 26 5 398 406 10.1159/000164276 18936543

[B9] Mioshi E Bristow M Cook R Hodges JR Factors underlying caregiver stress in frontotemporal dementia and Alzheimer’s disease Dement Geriatr Cogn Disord 2009 27 1 76 81 10.1159/000193626 19155621

[B10] Zarit SH Reever KE Bach-Peterson J Relatives of the impaired elderly: correlates of feelings of burden Gerontologist 1980 20 6 649 655 10.1093/geront/20.6.649 7203086

[B11] Dunkin JJ Anderson-Hanley C Dementia caregiver burden Neurology 1998 51 1 Suppl 1 S53 S60 9674763 10.1212/wnl.51.1_suppl_1.s53

[B12] Gauthier A Vignola A Calvo A Cavallo E Moglia C Sellitti L Mutani R Chio A A longitudinal study on quality of life and depression in ALS patient-caregiver couples Neurology 2007 68 12 923 926 10.1212/01.wnl.0000257093.53430.a8 17372127

[B13] Goldstein LH Atkins L Landau S Brown R Leigh PN Predictors of psychological distress in carers of people with amyotrophic lateral sclerosis: a longitudinal study Psychol Med 2006 36 6 865 875 10.1017/S0033291706007124 16490122

[B14] Nakagawa Y Uozumi T Tsuji S [Quality of life and burden in caregivers for ALS patients in Japan] Rinsho Shinkeigaku 2010 50 6 412 414 10.5692/clinicalneurol.50.412 20593668

[B15] Pagnini F Rossi G Lunetta C Banfi P Castelnuovo G Corbo M Molinari E Burden, depression, and anxiety in caregivers of people with amyotrophic lateral sclerosis Psychol Health Med 2010 15 6 685 693 10.1080/13548506.2010.507773 21154021

[B16] Chio A Vignola A Mastro E Giudici AD Iazzolino B Calvo A Moglia C Montuschi A Neurobehavioral symptoms in ALS are negatively related to caregivers’ burden and quality of life Eur J Neurol 2010 17 10 1298 1303 10.1111/j.1468-1331.2010.03016.x 20402747

[B17] Ringholz GM Appel SH Bradshaw M Cooke NA Mosnik DM Schulz PE Prevalence and patterns of cognitive impairment in sporadic ALS Neurology 2005 65 4 586 590 10.1212/01.wnl.0000172911.39167.b6 16116120

[B18] Witgert M Salamone AR Strutt AM Jawaid A Massman PJ Bradshaw M Mosnik D Appel SH Schulz PE Frontal-lobe mediated behavioral dysfunction in amyotrophic lateral sclerosis Eur J Neurol 2010 17 1 103 110 10.1111/j.1468-1331.2009.02801.x 19874396

[B19] Cedarbaum JM Stambler N Malta E Fuller C Hilt D Thurmond B Nakanishi A The ALSFRS-R: a revised ALS functional rating scale that incorporates assessments of respiratory function J Neurol Sci 1999 169 1–2 13 21 10540002 10.1016/s0022-510x(99)00210-5

[B20] Montes J Levy G Albert S Kaufmann P Buchsbaum R Gordon PH Mitsumoto H Development and evaluation of a self-administered version of the ALSFRS-R Neurology 2006 67 7 1294 1296 10.1212/01.wnl.0000238505.22066.fc 17030772

[B21] Wedderburn C Wear H Brown J Mason SJ Barker RA Hodges J Williams-Gray C The utility of the Cambridge behavioural inventory in neurodegenerative disease J Neurol Neurosurg Psychiatry 2008 79 5 500 503 10.1136/jnnp.2007.122028 17846114

[B22] Bozeat S Gregory CA Ralph MAL Hodges JR Which neuropsychiatric and behavioural features distinguish frontal and temporal variants of frontotemporal dementia from Alzheimer’s disease? J Neurol Neurosurg Psychiatry 2000 69 2 178 186 10.1136/jnnp.69.2.178 10896690 PMC1737062

[B23] Henry JD Crawford JR The short-form version of the depression anxiety stress scales (DASS-21): construct validity and normative data in a large non-clinical sample Br J Clin Psychol 2005 44 227 239 10.1348/014466505X29657 16004657

[B24] Zarit SH Orr N Zarit JM The hidden victims of Alzheimer’s disease: families under stress 1985 New York: New York University Press

[B25] Bédard M Molloy DW Squire L Dubois S Lever JA O’Donnell M The zarit burden interview Gerontologist 2001 41 5 652 657 10.1093/geront/41.5.652 11574710

[B26] Gundersen MD Yaseen R Midgard R Incidence and clinical features of amyotrophic lateral sclerosis in More and Romsdal County, Norway Neuroepidemiology 2011 37 1 58 63 10.1159/000329523 21860251

[B27] Huisman MH de Jong SW van Doormaal PT Weinreich SS Schelhaas HJ van der Kooi AJ de Visser M Veldink JH van den Berg LH Population based epidemiology of amyotrophic lateral sclerosis using capture-recapture methodology J Neurol Neurosurg Psychiatry 2011 82 10 1165 1170 10.1136/jnnp.2011.244939 21622937

[B28] Logroscino G Traynor BJ Hardiman O Chio A Mitchell D Swingler RJ Millul A Benn E Beghi E Incidence of amyotrophic lateral sclerosis in Europe J Neurol Neurosurg Psychiatry 2010 81 4 385 390 10.1136/jnnp.2009.183525 19710046 PMC2850819

[B29] Gibbons ZC Richardson A Neary D Snowden JS Behaviour in amyotrophic lateral sclerosis Amyotrophic Lateral Sclerosis 2008 9 2 67 74 10.1080/17482960701642437 18427998

[B30] Mioshi E McKinnon C Savage S O’Connor CM Hodges JR Improving burden and coping skills in frontotemporal dementia caregivers: a pilot study Alzheimer Dis Assoc Disord 2012 Epub ahead of print 10.1097/WAD.0b013e31824a7f5b 22354158

[B31] Trail M Nelson ND Van JN Appel SH Lai EC A study comparing patients with amyotrophic lateral sclerosis and their caregivers on measures of quality of life, depression, and their attitudes toward treatment options J Neurol Sci 2003 209 1–2 79 85 12686407 10.1016/s0022-510x(03)00003-0

[B32] Miyashita M Narita Y Sakamoto A Kawada N Akiyama M Kayama M Suzukamo Y Fukuhara S Care burden and depression in caregivers caring for patients with intractable neurological diseases at home in Japan J Neurol Sci 2009 276 1–2 148 152 18954877 10.1016/j.jns.2008.09.022

[B33] Goldstein LH Adamson M Jeffrey L Down K Barby T Wilson C Leigh PN The psychological impact of MND on patients and carers J Neurol Sci 1998 160 Suppl 1 S114 121 9851660 10.1016/s0022-510x(98)00209-3

[B34] Vignola A Guzzo A Calvo A Moglia C Pessia A Cavallo E Cammarosano S Giacone S Ghiglione P Chio A Anxiety undermines quality of life in ALS patients and caregivers Eur J Neurol 2008 15 11 1231 1236 10.1111/j.1468-1331.2008.02303.x 18803649

[B35] Renton AE Majounie E Waite A Simon-Sanchez J Rollinson S Gibbs JR Schymick JC Laaksovirta H van Swieten JC Myllykangas L A hexanucleotide repeat expansion in C9ORF72 is the cause of chromosome 9p21-linked ALS-FTD Neuron 2011 72 2 257 268 10.1016/j.neuron.2011.09.010 21944779 PMC3200438

[B36] DeJesus-Hernandez M Mackenzie IR Boeve BF Boxer AL Baker M Rutherford NJ Nicholson AM Finch NA Flynn H Adamson J Expanded GGGGCC hexanucleotide repeat in noncoding region of C9ORF72 causes chromosome 9p-linked FTD and ALS Neuron 2011 72 2 245 256 10.1016/j.neuron.2011.09.011 21944778 PMC3202986

